# Towards a common bean proteome atlas: looking at the current state of research and the need for a comprehensive proteome

**DOI:** 10.3389/fpls.2015.00201

**Published:** 2015-03-27

**Authors:** Sajad M. Zargar, Muslima Nazir, Vandna Rai, Martin Hajduch, Ganesh K. Agrawal, Randeep Rakwal

**Affiliations:** ^1^School of Biotechnology, SK University of Agricultural Sciences and Technology of JammuJammu, India; ^2^Department of Botany, Jamia Hamdard UniversityNew Delhi, India; ^3^National Research Centre on Plant Biotechnology, Indian Agricultural Research InstituteNew Delhi, India; ^4^Reproduction and Developmental Biology, Institute of Plant Genetics and Biotechnology, Slovak Academy of ScienceNitra, Slovakia; ^5^Research Laboratory for Biotechnology and BiochemistryKathmandu, Nepal; ^6^Organization for Educational Initiatives, University of TsukubaTsukuba, Japan

**Keywords:** nutrition, proteome atlas, common bean, human health, genomics

## Common bean, human health, and food security

The common bean (*Phaseolus vulgaris* L.) is a legume (Leguminosae; Cronquist, 1981) with high nutritional value that may be a critical food source in our battle to have food for all, i.e., food security worldwide (FAOSTAT, 2013). Nutrition-wise, the common bean is an important protein-rich, low-fat, nutrient-dense food containing high amounts of energy as carbohydrates. It is also rich in minerals (mainly Fe and Zn) (Pinheiro et al., 2010), and potentially disease preventing with health-promoting compounds exhibiting pharmaceutical properties (Hayat et al., 2014). Legumes, via their ability to fix atmospheric nitrogen, also play an important role in sustainable agriculture (Liu et al., 2011). Like other crops, the common bean bears the brunt of a changing climate resulting in diverse abiotic and biotic stresses which reduces its yield and nutritional status. These factors necessitate new research into its biology. An exponential increase in population size and current trends in bean consumption due to the presence of vital nutrients demands both its cultivation and high productivity in many parts of the world.

## Common bean genome research milestones at a glance

Improvement of the common bean means possessing in-depth knowledge of its genetic diversity, the genome and gene functions, to enable the analysis of pathways and networks in response to fluctuating environmental conditions. Various genomic resources for common bean are available and include physical maps, bacterial artificial chromosome libraries, anchored physical and genetic maps, expressed sequence tags, and the recently published complete genome sequence (O'Rourke et al., 2014; Schmutz et al., 2014). The 473 Mb genome sequence of common bean will help scientists to understand the evolution of the crop, synteny with other legumes and is a repository of genetic information for molecular breeders. Establishment of a 2.12-Mb transposon database for the common bean (www.phytozome.org), which includes 791 representative transposon sequences, will serve as an important resource for understanding genome evolution and genetic variation (Gao et al., 2014). In combination, a systems biology approach is required for in-depth analysis of the molecular substrates and target moieties regulating various metabolic pathways which is made possible by coherently integrating “omics” data. Here, we dwell upon the necessity for developing a common bean (*P. vulgaris*) proteome resource and thereby help translate this information into improving its nutritional value and to develop a more sustainable crop.

## Common bean proteomes

During past 10 years, common bean proteome studies have ranged from abiotic to biotic stress, seed to storage proteins, and diversity. Research in abiotic stresses have examined the effect of the gaseous pollutant ozone, revealing distinct protein changes in affected leaves by two-dimensional electrophoresis (2-DGE; Torres et al., 2007). Phosphoproteomic analysis revealed that enhanced phosphorylation of dehydrin plays a major protective role in the reversibility of cell wall extensibility during recovery from osmotic stress induced physical breakdown of cell wall structures (Yang et al., 2013). 2D-DIGE analysis showed the impact of drought stress on different biological pathways and molecular functions in leaves of drought tolerant and sensitive cultivars (Zadražnik et al., 2013). Response to chilling stress was dependent on length and manner of exposure to low temperature, as determined by divergent proteomic alterations in roots in response to varying periods of low temperature stress (Badowiec and Weidner, 2014).

Rust fungus infection of leaves revealed *R*-gene based defense modulates proteins similar to those in the basal system defense (Lee et al., 2009). Approaches have been developed to improve protein extraction from seeds with TCA–acetone followed by a clean-up step resulting in the highest amount of storage and defense proteins over a phenol method (De La Fuente et al., 2011). The TCA-acetone method for seeds in combination with 2-DGE analysis was applied to the analysis of improved common beans through the alteration of protein components (Natarajan et al., 2013). A further study explained the role of post-translational modifications of phaseolin proteins demonstrating phosphorylation during the transition from seed dormancy to an early germination stage (López-Pedrouso et al., 2014). Previously, seed proteomics had revealed that a lack of storage proteins leads to increased legumin, albumin-2, defensin and albumin-1, which contribute to elevated sulfur amino acid content and raffinose metabolic enzymes while simultaneously down-regulating the secretory pathway (Marsolais et al., 2010). A 2-DGE analysis revealed differences among cultivated and wild-type common bean cultivars as evidenced from differences in number as well as abundance of protein spots on gels, and used these factors to classify these cultivars based on their Centre of Domestications (COD; Mensack et al., 2010). Recently, a legume specific protein database (LegProt, http://bioinfo.noble.org/manuscript-support/legumedb) has been created which contains sequences of several legumes including the common bean. This resource significantly increases legume protein identification success rates and the confidence levels compared to the commonly used database NCBInr (Lei et al., 2011).

## Toward common bean proteome atlas: understanding global regulation

**Figure 1:**
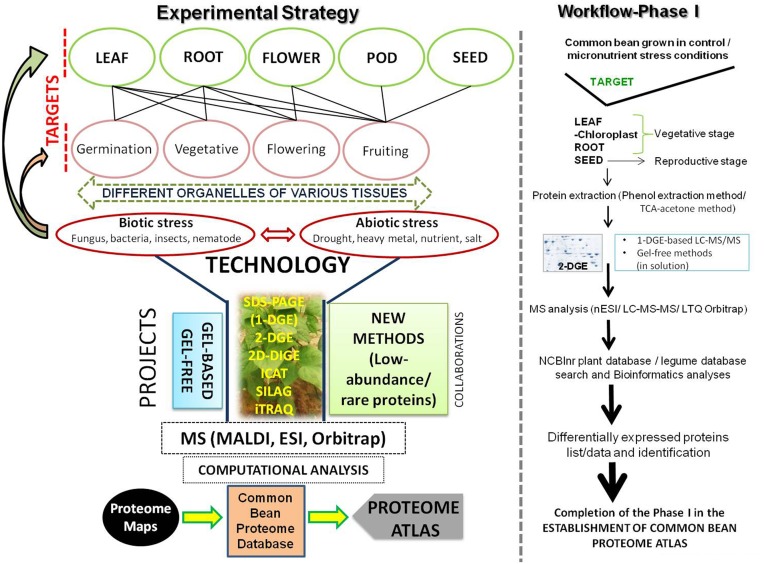
**Proteome atlas for the common bean – an overview**. Details are mentioned in the text.

These previous studies have all individually contributed to specific proteomes of the common bean. However, considering a focus on the improvement of the common bean, a comprehensive and systematic approach is required at the proteome level with an overall goal of creating a “PROTEOME ATLAS.” As such, studying the proteome from all the organs of the plant (leaves, stems, roots, flowers, pods, and developing and mature seed) under specific conditions (disease or other environmental conditions) and at particular developmental stages along with the organelle and secreted proteomes, will collectively help to identify unexplored pathways that can be utilized and targeted to address specific problems associated with this legume. For example, our group has developed a collection of diverse germplasm of the common bean which mainly includes landraces from Jammu and Kashmir, India (Zargar et al., 2014). Availability of landraces from other specific geographical locations from around the world will also deliver unique genetic stocks for valuable traits (e.g., abiotic or biotic stress tolerance). A big challenge will be the large number of experiments to be performed using both gel-based and gel-free approaches along with new methods for unraveling low-abundance/rare proteins. Figure 1 provides a snapshot of the work-plan, along with Phase I of the workflow as a first target for our research. Essentially, a particular trait and organ/tissue will be focused on under specific criteria to create a unique database (2D-map image and protein information) for that specific proteome. We are of the opinion that execution of this huge task will require collaboration among different laboratories that have expertise in various aspects of proteomic technologies. We intend to invite members of plant proteomics community through the INPPO platform (http://www.inppo.com/). Overall, proteomics linked with genomics and transcriptomics will likely enhance the agronomic merit as well as quality traits in the common bean by enabling us to first understand regulatory pathways and then enable the manipulation of these regulatory pathways to attain an improved and more sustainable crop.

### Conflict of interest statement

The authors declare that the research was conducted in the absence of any commercial or financial relationships that could be construed as a potential conflict of interest.
